# Hospital and Institutional News

**Published:** 1914-08-08

**Authors:** 


### HOSPITAL AND INSTITUTIONAL NEWS.

### HOSPITALS AND THE WAR.

So far, at the time of going to press, the hospitals have been chiefly affected by the declaration of war in three respects. They have had to face an immediate rise in prices, and to decide whether or not to lay in additional stores of food. They have already lost some off their medical staffs who have been called up, and their nursing staffs have been in some instances depleted by volunteers for active service. At the time of writing it appears that differences of opinion prevail as to the necessity of securing additional food supplies. Some hospitals began to purchase last week, some at present are content with their existing stores, and some have largely increased their orders. Among the latter may be counted especially pay hospitals and nursing homes. Several steam yachts have been offered to the Admiralty for hospital purposes, among which may be mentioned Lord Tredegar's steam yacht *Liberty* and Mr. C. H. Loeffler's *Albion*.

### THE BRITISH RED CROSS SOCIETY'S PREPARATIONS.

After a meeting of the Council of the British Red Cross Society on Tuesday, it was understood that the Society was in a position to meet any likely emergencies. Lord Rothschild presided at this meeting, and among those present were Mr. G. H. Makins, C.B., F.R.C.S., and Sir Frederick Treves. A public appeal is probably to be issued for funds, but what the policy of the Society will be must depend upon eventualities which, from the nature of the case, may change from day to day. The Society will have its hands full in this country whether or not it despatches nursing corps to the Continent. The general work of organisation is rapidly proceeding, and as we go to press a further meeting is being held, and sub-committees are being appointed to deal with the different aspects of the organisation.

### SURGEONS' SERVICES IN REQUEST.

Medical practitioners who are civilians and desire to serve at home or abroad should communicate at once with the Secretary, War Office. Gentlemen who are accepted for such service will be granted the temporary rank of lieutenant, and must fulfil the following conditions:—(1) They must be registered practitioners. (2) They must engage for a period of twelve calendar months or until their services are no longer required, whichever shall first happen. (3) Their pay to be twenty-four shillings a day, inclusive of all money allowances, except travelling allowances and expenses when travelling on duty. (4) They will be granted free passages to and from any country abroad to which they may be sent. (5) They must devote their whole time to military service. (6) They will receive a gratuity of £60 at the termination of their engagement if they have rendered satisfactory service. (7) They should not exceed thirty-five years of age, but in exceptional circumstances gentlemen between thirty-five and forty may be accepted. (8) They must furnish: (*a*) A medical certificate showing that they are in good health, of sound constitution, and fit for hard physical work. (*b*) A statement of any previous service with the Army. (*c*) A recommendation as to character from a person of position and responsibility. (*d*) A certificate as to age.

### ATTENDANTS "AN UTTERLY UNSKILLED CLASS."

According to the local press, Mr. David Davis, who last week submitted the supplemental report of the Asylums Committee to the Birmingham City Council, is credited with the extraordinary statement that "the nurses and attendants were an utterly unskilled class." What is no less surprising in a city which he himself described as being amongst the first three as to mental hospitals in the country and as to the hours of work and wages paid, no comment apparently was made by anyone present at the meeting. No explanation was asked as to this wholesale condemnation of one class of institutional workers, and the stigma was accepted without protest; but we cannot believe that either the medical superintendent at Winson Green or the attendants referred to will let this sweeping condemnation pass without immediate challenge. The Lord Mayor, Lieutenant-Colonel Martineau, who presided, with Mr. Davis must be held at once to account for an assertion that can only undermine discipline and fill with just resentment every officer in the mental institutions under the Asylums Committee's control.

### THE SUPPLY OF DRUGS BY POOR-LAW DOCTORS.

We are glad to see the Local Government Board setting its face against the bad old practice of medical officers to Poor-Law Unions being compelled to supply the drugs needed for the sick out of their salaries. At a meeting of the Keynsham Board of Guardians last week, with Mr. F. R. Morse, the chairman, presiding, a letter was read from the Local Government Board with reference to the raising of the salary of Mr. C. Harrison, medical officer to the institution, from £60 to £85 per annum. The Board stated that they would "not offer objection to the increase proposed, but they suggest that the Guardians should take this opportunity of reconsidering the matter of this officer's remuneration, with a view to the adoption of an arrangement whereby all medicines required for the use of the sick inmates would be provided at the cost of the Guardians and not at that of the medical officer." On this the clerk remarked that a properly-equipped surgery would mean a considerable outlay, others expressing the opinion that the institution was too small. The latter view, on the motion of the Chairman, prevailed; but this unfortunately does not alter the fact that the principle of a medical officer having to supply drugs out of his own pocket is thoroughly vicious, and is condemned because it makes the question of the prescriber's own pocket enter into his mind in which the patient's requirements should alone have influence. We have no doubt that before long this evil system will be as formally abolished in practice as it is discouraged in theory; and the sooner the better for the sick poor and medical officer alike.

### INCREASED SALARIES UNDER THE ASYLUMS BOARD.

A special sub-committee of the Metropolitan Asylums Board have been considering the question of the salaries of the medical staff and the terms of their appointment. The officers have from time to time approached the Board with a view to removing some of the disadvantages under which they suffer, and although the Board has not as yet decided to accede to all the requests for readjustment, they have allowed certain modifications which for a time at least may remove some of the grievances. With regard to the remuneration the Board have decided to adopt a more liberal scale. The present salaries of medical superintendents at the institutions is £600 a year, rising by annual increments of £50 to a maximum of £800, whilst the salary of the medical superintendent at Darenth Industrial Colony with Bridge Industrial Home is £700, rising to £900; all with unfurnished house and washing. Henceforth the salaries are to be for Leavesden, Caterham, and Tooting Bec Mental Hospitals £725, rising by annual increments of £50 to a maximum of £925; Edmonton Mental Hospital £625, rising to £825; and Darenth Industrial Colony with Bridge Industrial Home £825, rising to £1,025, to be reduced £100 in the event of the officer ceasing to undertake work in connection with the Home. The salaries of the assistant medical officers have been revised, and in future will be as follows: Senior, mental hospitals, £350, rising by annual increments of £25 to a maximum of £450; hospitals £350, rising to £400; and the children's hospitals £350, rising to £400. Second assistants, mental hospitals, £300, rising to £350, and the hospitals and children's hospitals £300. Junior assistants at all the institutions will receive £250 a year.

### THE ASSISTANT MEDICAL OFFICERS' VIEWS.

Objection had been taken by the Association of Hospital Assistant Medical Officers in the Board's service to the title of "assistant medical officer," but the medical superintendents in both services agreed that the title accurately describes the position of the officers concerned, though they suggested that the title "resident medical officer" might be adopted; but the sub-committee could not see sufficient reason for making an alteration, as it was well understood in the medical service, and had no detrimental effects on their future appointments. The question of emoluments and their value was also considered, and it was decided that the total value should be increased to £100 per annum. Food was another matter of which serious complaints were made, the officers stating that they were not supplied with "first quality food," and that the cooking and attendance was unsatisfactory at many of the hospitals. There were good grounds for the complaint, the sub-committee ascertained, and suggested that complaints should be considered by the various committees. A report on the diet, making many important suggestions, had been prepared by the medical superintendents and the matrons, which it was hoped would lead to acceptable improvements being made, more especially as they affected the variety of food prepared. The time off duty was alleged to be insufficient, but the sub-committee could not see their way clear to extend it, as the superintendents stated that it was "ample."

### THE "TONE" OF THE ASSOCIATION.

The sub-committee, in conclusion, informed the Board that they could not profess to have solved the problem of satisfying every demand upon the Board; indeed, in some of the matters complained of it was difficult to see how perfection could be secured. "We do not know," the sub-committee added, "to what extent the members of the Association of Hospital Assistant Medical Officers have approved of the tone which characterises some of the communications addressed to the Board, a tone which has not been emulated by any other body of officers who have appealed to the Board or by any union which has approached the Board. Moreover, we feel it incumbent upon us to point out that in some of these communications the manner in which the complaints are set out on behalf of the assistant medical officers gives an erroneous impression of the Board's attitude. Isolated cases are made to appear as general rules, some matters of fact are incorrectly stated, information for the cause of the Board's action which would put a different complexion on some of the points is not given, and ancient history has been searched for some small points which are given undue importance. We have, however, given careful consideration to the points placed before us on behalf of the assistant medical officers, and believe that if the Board adopt the proposals we have put forward they will have . . . done what reasonably can be expected of them to maintain their medical service on a satisfactory basis."

### THE TUBERCULOSIS SCHEME FOR LONDON.

Particulars of the London County Council scheme for the treatment of tuberculosis have already been published in The Hospital, and they have now been approved by the Local Government Board subject to various criticisms as to detail. The Board, like many people who have had the scheme under consideration, regret that the County Council have not been prepared, as the Councils of other great towns have been, to undertake to provide the accommodation necessary for the whole community, insured and uninsured alike. They express some doubt as to whether the proposals in regard to the selection of cases for residential treatment will prove satisfactory in actual working. While the Board take no exception to the establishment of the proposed honorary advisory board, they consider that the Council, instead of authorising this board to appoint medical referees for the purpose of examining selected cases recommended for residential treatment, would have been well advised to appoint a clinical tuberculosis officer whose duties would have included advising the Council in the selection of cases for residential treatment. It further appears to them that the proposed system of payment by fee per case is not generally desirable as part of a permanent arrangement. The Board approved generally of the proposals as regards adults on the understanding that the provision of beds at present proposed is to be regarded as an instalment only of what will ultimately be required. In regard to the proposals for the treatment of children the Board are now in communication with the Board of Education.

### A PATIENT'S INGENUITY.

It is not often, writes a correspondent, that we find a patient making his own surgical appliances, but we have recently heard of a case where a carpenter who, having undergone an amputation above the knee, was supplied in due course with an artificial limb, but suffered so much discomfort from its fit that he decided to make one for himself; this he did most satisfactorily, and to-day always wears the "home-made" limb. It is of interest to note that the leg is excellently modelled, jointed at knee and instep, and is made of leather and wood.

### AN EMPLOYERS' PLAN FOR RAISING SUBSCRIPTIONS.

A remarkably practical form of appeal has been issued by certain manufacturers in the area served by the Wolverhampton General Hospital. It is original both in conception and in the manner in which it is carried out. Before describing its features, the circumstances out of which it arose may be stated briefly. The income of the hospital for the past ten years has virtually stood still. It has failed to bridge the difference between £10,300 and £11,000. In the meantime the expenditure has advanced by £3,000. The circular which certain firms have issued, not to the public—though it has been published, we understand, as an example to other sections of the trading community and employers of labour—but to other manufacturers, summarises the financial condition in a four-line table, and then proceeds as follows: "In an endeavour to prevent such a contingency [the closing of wards], we have increased our annual subscription, and hope you may be able to help in a similar manner the effort which is being made by the board of management." The circular concludes with a list of the firms signing it, against whose signatures appears the amount of their subscription in 1913, and the sum to which it has been increased in this. In every case but two the amount is doubled, and in one it is raised from one guinea to five. There are seventeen firms in the list of signatures, which includes seven who have not previously subscribed, and the result is that the sum total has been raised from £33 to £95, an extremely creditable result. It will be noticed at once that an appeal which records a success, and gives chapter and verse for it, awakens more interest (it is more novel) than one which describes needs and difficulties. It is obvious at once that there is no reason why the list should be limited to seventeen, and now that the ball has been set rolling, we hope that the number participating in this effort will increase, and that other groups of employers will be persuaded to adopt the same plan. We also commend this method of appealing to other institutions as worthy of being introduced into their own neighbourhoods.

### SECRETARIAL CHANGES AT LLOYD HOSPITAL, BRIDLINGTON.

Owing to the illness of Mr. B. Heselton the Lloyd Hospital, Bridlington, has been deprived of the services of its honorary secretary and treasurer, which he has carried out for a long period. The new honorary secretary is Mr. T. E. Dufty, who has undertaken the duties of this office, and will no doubt be engaged upon securing the increased subscriptions which were asked for by the president, Mr. Y. G. Lloyd-Greame, at the recent annual meeting. There is an adverse balance of approximately £400, which it is important should not be allowed to accumulate; and now that annual subscriptions, whether in the form of working-men's regular collections or equally from private individuals of the middle and richer classes, are recognised as the only permanent means of providing a stable hospital income, it is to be hoped that they may be found in increased numbers from this district. Annual subscriptions, indeed, are the only practicable alternative to endowment, and a living bond of interest between supporters and hospitals can best find its practical expression in this way. Mr. T. E. Dufty's first year of office will be marked, we hope, by an increase in this respect.

### THE WEST END HOSPITAL AND REBUILDING.

Lord Hindlip has been appointed chairman of the West End Hospital for Diseases of the Nervous System, in succession to Mr. Leonard Brassey, M.P., who has resigned. Mr. Brassey intends to reside chiefly at his country seat, and in future will not be so much in London. He therefore feels unequal to guiding the policy of the hospital under present circumstances. However, he retains a seat on the committee, and has made, we understand, a most generous proposal in respect of the rebuilding of the hospital.

### COTTAGE HOSPITAL PAY-WARDS.

Striking evidence of the usefulness of pay-wards is forthcoming from the recent annual report of Wellingborough Cottage Hospital. The extensions which were carried out a short time ago have already achieved success, and the ward reserved for paying patients has brought in a revenue of £84 in three months, having been used by the patients during this period. While the town employees' fund and the Midland Railway fund showed unfortunate decreases, the town people's subscriptions realised £546 apart from special contributions, and the annual fête has mounted up to nearly £200 this year. It would be interesting to know the detailed history of this pay-ward, how the question of fees is arranged, and what opposition, if any, the scheme first met with. Other cottage hospitals would be interested in these facts, for though most of them receive from time to time patients from whom payments are exacted, pay-wards as such are not so common, and the details of their working are therefore of interest. If the Secretary of Wellingborough Hospital cares to send us the details of this departure we shall be glad to publish them for the benefit of other institutions which may be contemplating a similar step.

### THE DEWSBURY INFIRMARY ENDOWMENT FUND.

The trustees of the Dewsbury Infirmary recently prepared a scheme for the settlement of the Endowment Fund of the institution, which has been adopted by a special meeting of subscribers. The new scheme takes the form of a deed stating in what securities money left to the infirmary might be invested, and applies to the various funds which have been credited to the Endowment Fund and hitherto been invested simply on the initiative of the board of management. Such a scheme, no doubt, presents itself not merely as a formal and businesslike way of doing things, but also as relieving the trustees and those charged with the investment of the hospital's funds of an unnecessary responsibility, by enabling them to have the benefit of formal guidance. There was no opposition to the scheme. On the same occasion an appeal was made for £70 in connection with the starting of the new departments for eye, ear, nose, and throat complaints. As a substantial sum was received through the Alexandra Day collections, it is to be hoped that local interest has been sufficiently aroused to give the new departments the financial send-off which they need.

### SUGGESTED EXTENSIONS AT SWANSEA HOSPITAL.

The Building Committee having approved the plans for the north wing extensions at Swansea Hospital, it has been decided, on approval by the Finance Committee, to proceed with the work at the eye department, where additions to the extent of £1,000 are in hand. But a larger project is on foot—namely, to carry out the long contemplated ward of twenty-four beds above the Llewelyn Ward, to renovate the latter at a cost of £1,250, and to secure comfortable servants' accommodation. The new ward would cost £5,000, and the money to pay for it and the other alterations would come out of a legacy of £10,000 which has been left by the late Mr. Graham Vivian on condition that a ward be named after him. Unfortunately the overdraft on the building account is still £6,000, and therefore a month's consideration is being given to the proposed scheme. To pay off that overdraft and then to appeal for the balance required for the new developments would seem the most statesmanlike way of dealing with both. It would also appeal most to the public interest.

### THE RALLY OF THE HOSPITAL SATURDAY FUND

It is officially stated that the Hospital Saturday Fund for London is recovering with ease and rapidity from the set-back caused by the operations of the National Insurance Act. The receipts up to July 31 amounted to £11,975, as compared with £10,935 for the corresponding period of last year—an increase of over £1,000. It is remarkable that the operations of the Insurance Act have caused a noticeable increase in the demand for dental treatment, surgical appliances, accommodation at convalescent homes, and treatment in sanatoria and hospitals. The subscribers to the Fund now realise that these demands can be met by this Fund, and that only in the case of sanatorium benefit for insured persons can they be met under the provisions of the Insurance Act. One tribute to the Act is that it has revealed an amount of hitherto untreated illness which, while creating difficult problems, is of service in calling attention to the extent of the ill-health for which no previous treatment has been available.

### DR. ARTHUR NEWSHOLME AND SANATORIA.

In opening the Tuberculosis Hospital of the Eastbourne Corporation last week, Dr. Arthur Newsholme, medical officer of the Local Government Board, declared that it meant an approach towards the completion of a well co-ordinated plan for the treatment of disease in the town. If the provision for tuberculosis in any area were still inadequate, he added, it was because local authorities had not realised that half of their local annual expenditure on the treatment of non-insured tuberculous patients, as well as one-half of any deficit in the cost of treating insured persons, could be recovered from national funds. The responsibility for promoting schemes rested with the local authority. The importance of housing as a means of diminishing tuberculosis he fully realised, and the first essential was to separate the sick from the healthy, and further to arrange for the adequate treatment of the sick and the proper comfort of advanced cases. But since the latter was practically impossible in the homes of the wage-earning classes, in the interest of the relatives of the sick person as much as on his own behalf, greatly increased institutional accommodation was necessary. But improved housing conditions and hospital accommodation were probable in a few years, especially for consumptives, and he congratulated Eastbourne on making a beginning. It is curious to note that the sanatorium, against which there has been a reaction during the past few years, is once more surviving criticism, and lasting in a way in which none other form of treatment has done so far. In the present instance, having found out that many early cases cannot satisfactorily be got at by tuberculosis dispensaries, nor be rescued by domiciliary treatment, a cry is raised that the housing question must be tackled on a national scale if the "campaign against consumption" is to be really more than a paper success. By this is meant that residential conditions must form part of an adequate treatment, and that is forcing us back again on the sanatorium as the one place in which they can be medically controlled. There must be some permanent value in an institution which always reappears as an inevitable part of tuberculosis therapeutics.

### THE EXTENSION OF ALTRINCHAM HOSPITAL.

By having increased its accommodation to some sixty beds the Altrincham Hospital has been re-opened in almost a new form, with the enhanced prestige of having raised by subscription the necessary £11,500, which the alterations, additions, and improvements have cost. Mr. Lindsell, chairman of the board of management, thus sees a new phase of development in front of his institution, while the support he has received in effecting the change should justify the expectation that the increased cost of maintenance will be found without undue effort. A resident house surgeon is being appointed, and the institution has taken on a new importance which should gain it new supporters in the district.

### HOSPITAL SCHOOLS.

Education and medical treatment are going increasingly more closely together: teachers are being expected to give instruction in hygiene and elementary physiology, and institutions for sick children, especially for "cripples," are having to provide educational facilities. The marriage of medicine and education is seen in the newly opened Leasowe Sanatorium, established by the Liverpool Invalid Children's Aid Association for poor children suffering from surgical tuberculosis. The Association has applied to the Treasury for a grant in connection with the building. The ambitious scheme will be completed only when two additional blocks to the two now opened are finished and ready; and the site extends to fourteen acres in the Wirral Peninsula, some 250 yards from the shore at Leasowe. Mr. T. W. Haigh, architect of the new children's infirmary in Liverpool, has drawn the plans, which comprise four hospital blocks, each of which accommodates forty-four children, an administrative block with an operating theatre and the resident staff's quarters, together with an $x$-ray room, and, as will be expected, a school-room. There are also observation wards. The Board of Education, through whom the grants are paid, has endorsed the approval of the plans given by Dr. Hope, the medical officer of health for Liverpool. With the first two blocks now open, the house at present on the site is serving as the temporary quarters of the staff; and beds are already allotted to neighbouring authorities. Of the eighty-eight beds now available sixty-six or rather more are now in occupation. One of the wards in use is curious from the fact that its scheme of decoration was dictated by an anonymous donor of £1,000, who wished a scheme of violet colour to be carried out, so from wall decorations to flower vases and children's jackets violet is everywhere seen. Of the £44,000 at which the total cost has been estimated £17,000 has been received from the Treasury. There are about half a dozen institutions of this kind in the country at present, and the Leasowe Sanatorium is not only the latest but likely also to be the best.

### INSTITUTIONAL CO-OPERATION.

It is therefore a striking example of the success which attends the combination of State aid with voluntary enterprise, and represents not merely a personal triumph for the Liverpool Invalid Children's Aid Association, which has been quick to seize, or rather to create, an opportunity, but also for the spirit of co-operation, which we have insisted on so often as the only statesmanlike solution of the hospital problems of the day. In Sir George Newman, chief medical officer of the Board of Education, and in Miss Margaret Bevan, the honorary secretary, the combination of the voluntary spirit and of State aid is happily symbolised.

### AN IMPROVEMENT AT HANWELL MENTAL HOSPITAL.

The Mental Hospitals Committee of the London County Council, acting on the principle that as far as possible all cases of pulmonary tuberculosis in a mental hospital should be segregated, has decided to carry out an improvement at Hanwell Mental Hospital. It is intended to convert No. 25 Ward into a phthisical ward for female patients, and this will involve the provision of a shelter on the south side of the day room, the alteration of all the windows to make them open to their full extent, the installation of additional steam-heating apparatus to increase the ventilation through the roof, and the substitution of unglazed lattice-work casement for windows, shutters and frames in the single rooms. The cost of the adaptation is estimated at £220, and the work is to be put in hand immediately.

### THE DOCTOR IN NATIONAL EDUCATION.

The President of the Board of Education, Mr. J. A. Pease, in a speech in the House of Commons on the education vote last week, summarised the progress that had been made in the medical side of the work. He said he regarded the health of the children as a central preliminary of their education. Medical men to the number of 1,097, 84 women, and 300 specialists were now engaged in inspecting children. Half the children in the schools were found to be in need of dental treatment. In two years the sum spent on medical service had grown from £47,000 to £130,000, and in the present Estimates £175,000 was set down. He had been able to secure increased grants for the 365 special schools for mental and physical defectives, and the present provision of open-air schools was inadequate: there were only 945 such places, and he was informed by his medical advisers that half a million children were in actual need of them. He was satisfied, however, that we were behind no other country in the provision made for physical exercises. Provision for feeding 358,000 children was being made, and where the work was well done he hoped, by a supplementary grant of

£77,000, to contribute half the cost to local authorities. He added that 200 schools for mothers were in existence, and also that while recognising the difficulties, he could not promise a long life to these schools in thickly-populated centres which were not prepared to provide some kind of playgrounds. He hoped next year to include £50,000 in the Estimates to assist local authorities. This is enough to show the increasing part which medical work is playing in national education, and it must be remembered that the more the work grows the more it will grow. For one of the best results of a great medical service is the unpleasant one of revealing an extent of need that was never previously suspected.

### BATH GUARDIANS URGED TO REBUILD.

At the meeting of the Bath Board of Guardians held on July 29 the report of the Joint General Purposes and House Committee (which report was ultimately adjourned until September) was received. The report recommended that Mr. Saxon Snell should be instructed to prepare the conditions for and to act as an assessor in a competition for plans to provide a complete hospital for 106 beds. The adoption of this report would, of course, necessitate the rescinding of the resolution of April 22 deferring the consideration of building a new infirmary for twelve months. In view of the Guardians' recent attitude it is worthy of note that during the discussion relating to the committee's report mention was made of a report of the master, dated July 1, 1913, in which he expressed the opinion that the prejudice of the poor to the workhouse and infirmary was dying out. He went on to point out that the buildings, except the Jubilee and maternity wards, were old and straggling, that there was no means of isolating children, and that the bathing and lavatory accommodation was inadequate in certain of the men's and women's wards. He also pointed out that a lift was necessary, since the dead had to be removed through other wards to the central staircase, in some cases passing through four wards. How impossible it is, therefore, for any public-spirited body to acquiesce in further delay is abundantly evident from the statements of their own lay officer, as reported to us above.

### SANATORIUM PATIENTS AND SICK PAY.

Among the questions which have arisen in connection with sickness benefit is whether payment of it should be made to the patient while he is undergoing sanatorium treatment. In many cases it appears that when an insured person enters a sanatorium payment of sickness benefit goes first to the committee, and then if he has no dependants to the general purposes fund. In other words, if he has no dependants it means that he loses his sick pay. But the same rule does not apply supposing that an insured person becomes a patient in a hospital, convalescent home, or workhouse, for in these cases the sick pay is accorded to him. Various insurance committees have regarded this disparity of practice as arbitrary and anomalous, and as an instance we may note that the Birmingham Insurance Committee has lately passed a resolution to the effect that the payment of sickness benefit to an insured person in a sanatorium be approved. The Birmingham Committee, in fact, felt that sanatoria patients should not be penalised in this respect, and they have approached various other committees with a view of getting them to endorse their opinion. The Norfolk Insurance Committee has adopted a similar view, and it is surely desirable that the sanatorium, which so far has been the scapegoat of the institutions connected with the Insurance Act should avoid penalising patients in this way, so long as other institutions do not do so. However, just the claim of an institution on an insured patient with no dependants may be, a uniform practice is most desirable.

### IMPORTANT BEQUESTS TO BRIGHTON HOSPITALS.

Mr. Charles Chetwode Baily, of Brighton, who was largely interested in the Rock Brewery, Brighton (Limited), and died in Aberdeen at the end of May, has left £10,000 to the Inalienable Fund of the Royal Sussex County Hospital, Brighton; £5,000 to the special Endowment Fund of the Royal Alexandra Hospital, Brighton; and £2,000 to the Endowment Fund of the London Hospital. As regards each of these bequests he requested (but created no trust in the matter) that each institution should name a bed or cot after him. He also left £1,000 each to the Endowment Fund of the Brighton, Hove, and Preston Dispensary; the Royal Hospital for Incurables, Putney Heath; and to the Surgical Aid Society. He also left £10,000 upon trust for his cousin Eleanor Hyacinthe Mundy and her issue, whom failing, to the Inalienable Fund of the Royal Sussex County Hospital, Brighton. These bequests are worth recording, for, on the whole, the Southern County Hospitals outside London do not come in for such large sums as those received by the great industrial centres of the Midlands and the North.

### THE CRISIS AND THE DRUG MARKET.

As a result of the European crisis the Drug market is entirely disorganised, and neither buyers nor sellers know what to do. As this country is so largely dependent upon the Continent for its supply of drugs, prices are bound to advance considerably, and in some cases enormously. In the circumstances, it is not surprising that business is practically at a standstill. Such drugs as quinine, carbolic acid, opium, chloroform, morphine, iodine, iodides, mercurials, etc., are always affected by wars as a result of an increased demand; but there is apparently no limit to the list of articles which will be scarce and dear while the present crisis lasts. Although buying on a large scale is out of the question, there should be little difficulty in ensuring a sufficient supply of essential drugs to meet immediate requirements; and, while there appears to be no ground for alarm, it is obvious that care should be taken to prevent waste and to discourage extravagant prescribing.

